# Supplementary material for: Inhibition of autophagy-lysosomal function exacerbates microglial and monocyte lipid metabolism reprograming and dysfunction after brain injury
Source: bioRxiv. 2025 Sep 8:2025.09.04.674092. Preprint. [Version 1] doi: 10.1101/2025.09.04.674092 (PMC12439913; doi:10.1101/2025.09.04.674092)
Supplement: Supplement 8 [file NIHPP2025.09.04.674092v1-supplement-8.pdf]

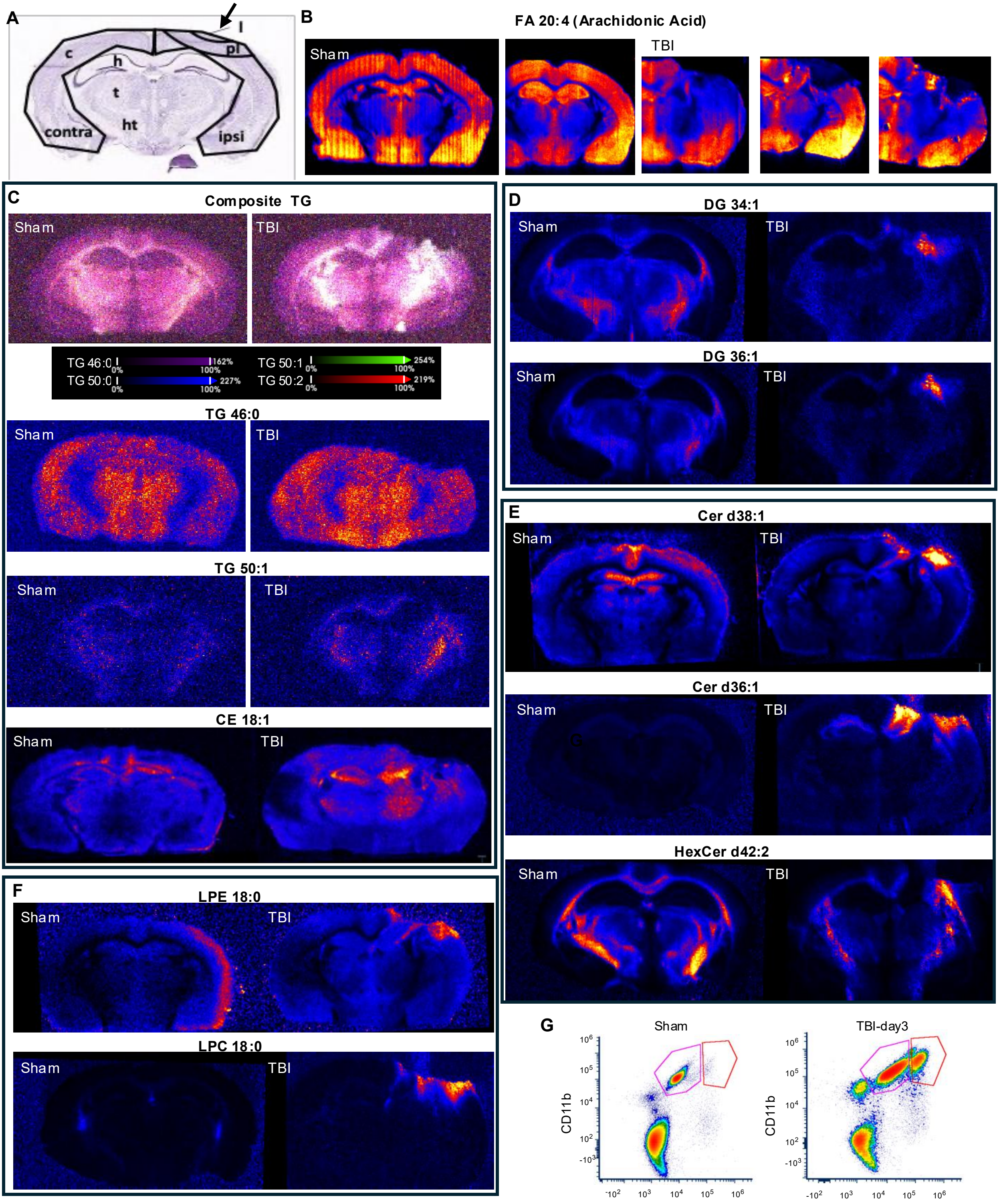
A
c
h
t
ht
contra
ipsi
pl
l
B
FA 20:4 (Arachidonic Acid)
Sham
TBI
C
Composite TG
Sham
TBI
TG 46:0 0% 100% 162%
TG 50:0 0% 100% 227%
TG 50:1 0% 100% 254%
TG 50:2 0% 100% 219%
TG 46:0
Sham
TBI
TG 50:1
Sham
TBI
CE 18:1
Sham
TBI
D
DG 34:1
Sham
TBI
DG 36:1
Sham
TBI
E
Cer d38:1
Sham
TBI
Cer d36:1
Sham
TBI
HexCer d42:2
Sham
TBI
F
LPE 18:0
Sham
TBI
LPC 18:0
Sham
TBI
G
Sham
TBI-day3
CD11b
CD45
Figure S1

Figure S1. TBI leads to accumulation of neutral lipids in microglia and infiltrating macrophages. Related to Figure 1. (A) Schematic representation of a coronal mouse brain section illustrating spatial terminology used in this study. The ipsilateral hemisphere (ipsi) refers to the side of the brain directly impacted in the controlled cortical impact (CCI) model of TBI (lesion site (l) is indicated with arrow), while the contralateral hemisphere (contra) is the uninjured side. The perilesional (pl) cortex (c) denotes the cortical region immediately adjacent to the lesion core (above hippocampus (h)) representing tissue at risk for secondary injury and a key area of immune and metabolic response. This schematic serves as a reference for the anatomical orientation of tissue punches, imaging regions, and data interpretation throughout the manuscript. Thalamus (t), hypothalamus (ht). (B-F) Desorption Electrospray Ionization Mass Spectrometry Imaging (DESI-MSI) of coronal mouse brain slices, sham vs TBI day 3 showing spatial distribution of different lipid species. (B) Arachidonic acid (FA 20:4) in two sham (left) and three TBI (right) animals demonstrating reproducibility. (C) Neutral lipids including composite of four triglyceride (TG) species (top), two of the component TG (TG 46:0 and TG 50:1) and cholesteryl ester (CE 16:0). (D) Diacyl glycerols (DG 34:1 and DG 36:1). (E) Ceramides (Cer d38:1 and Cer d18:1/18:0) and hexosylceramide (HexCer d42:2). (F) Lyso-phospholipids including lysophosphatidylethanolamine (LPE 18:0) and lysophosphatidylcholine (LPC 18:0). (G) Flow cytometry scatterplot demonstrating gating strategy for identifying microglia (CD11B+CD45int) and monocytes (CD11B+CD45hi) in sham (left) and TBI (right).

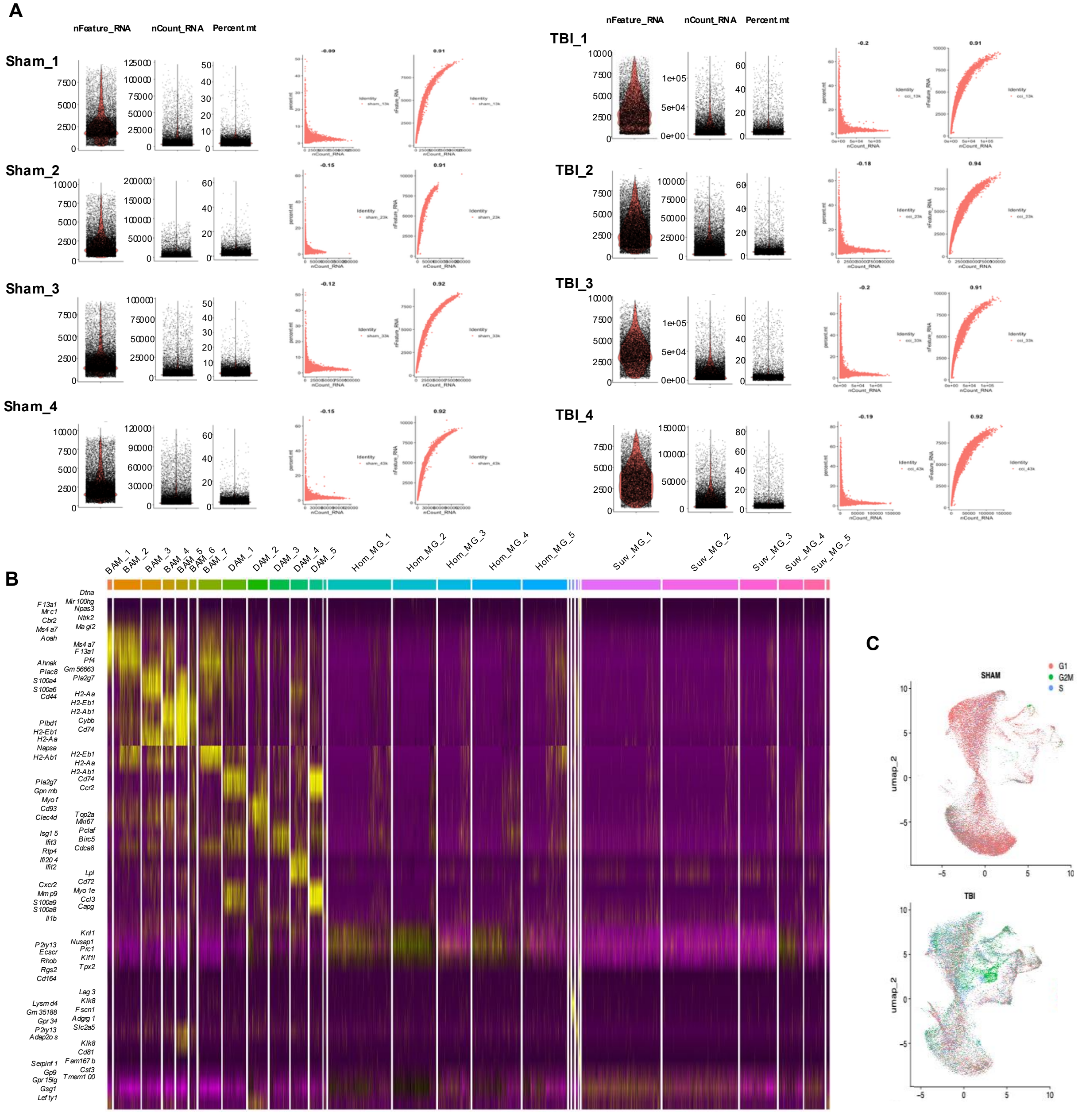
A
nFeature_RNA
nCount_RNA
Percent.mt
Sham_1
Sham_2
Sham_3
Sham_4
TBI_1
TBI_2
TBI_3
TBI_4
nCount_RNA
Identity
B
BAM_1
BAM_2
BAM_3
BAM_4
BAM_5
BAM_6
BAM_7
DAM_1
DAM_2
DAM_3
DAM_4
DAM_5
Hom_MG_1
Hom_MG_2
Hom_MG_3
Hom_MG_4
Hom_MG_5
Surv_MG_1
Surv_MG_2
Surv_MG_3
Surv_MG_4
Surv_MG_5
Dtna
Mir100hg
Npas3
Ntrk2
Magi2
Ms4a7
F13a1
Pf4
Gm56663
Pla2g7
H2-Aa
H2-Eb1
H2-Ab1
Cybb
Cd74
Ccr2
Top2a
Mki67
Pclaf
Birc5
Cdca8
Lpl
Cd72
Myo1e
Ccl3
Capg
Knl1
Nusap1
Prc1
Kif11
Tpx2
Lag3
Klk8
Fscn1
Adgrg1
Slc2a5
Cd81
Fam167b
Cst3
Tmem100
F13a1
Mrc1
Cbr2
Ms4a7
Aoah
Ahnak
Plac8
S100a4
S100a6
Cd44
Plbd1
H2-Eb1
H2-Aa
Napsa
H2-Ab1
Pla2g7
Gpnmb
Myof
Cd93
Clec4d
Isg15
Ifit3
Rtp4
Ifi204
Ifit2
Cxcr2
Mmp9
S100a9
S100a8
Il1b
P2ry13
Ecscr
Rhob
Rgs2
Cd164
Lysmd4
Gm35188
Gpr34
P2ry13
Adap2os
Serpinf1
Gp9
Gpr15lg
Gsg1
Lefty1
C
SHAM
TBI
G1
G2M
S
umap_2

Figure S2

Figure S2. TBI causes pronounced lipid metabolism reprogramming in microglia and macrophages. Related to Figure 2. (A) Quality control plots for each of sham 1-4 (left) and TBI 1-4 (right) samples used for scRNA-seq analyses. Left – three violin plots visualizing nFeature_RNA, nCount_RNA, and percent.mt QC metrics. Right – two FeatureScatter() generated plots of nCount_RNA vs perent.mt and ncount_RNA vs nFeature_RNA showing overall complexity and mitochondrial stress across cells and samples.

Together, these panels confirm the technical consistency and biological comparability across the four individual samples in both the sham and TBI groups. All samples underwent identical QC filtering thresholds, and no individual replicate displaying outlier behavior in these metrics. (B) Heatmap displaying top 5 differentially expressed genes for each cluster, selected based on highest average log fold-change using Seurat's FindAllMarkers() function. Gene expression values were scaled across cells and centered to highlight relative expression patterns. Each column represents a single cell, grouped by cluster identity, and each row corresponds to a marker gene. This visualization highlights cluster-specific transcriptional signatures, enabling annotation of distinct cell types and states. Marker genes were selected using a Wilcoxon rank-sum test with an adjusted p-value < 0.05 and minimum expression threshold across cells. Color scale indicates z-scored expression levels. (C) Cell cycle state classification of single-cell transcriptomes from sham and TBI cortex. Uniform Manifold Approximation and Projection (UMAP) plots showing cells color-coded by predicted cell cycle phase (G1, S, or G2/M) using Seurat's built-in cell cycle scoring pipeline. Cell cycle phases were inferred based on canonical marker gene expression profiles. The analysis reveals a higher proportion of TBI-associated cells in S and G2/M phases, consistent with overall increased proliferative activity. This shift explains the observed elevation in nFeature_RNA values after TBI in panel A, and supports biological findings from downstream pathway enrichment analysis, which identified cell division-related terms as the most significantly upregulated in the TBI condition (Figure 2E).

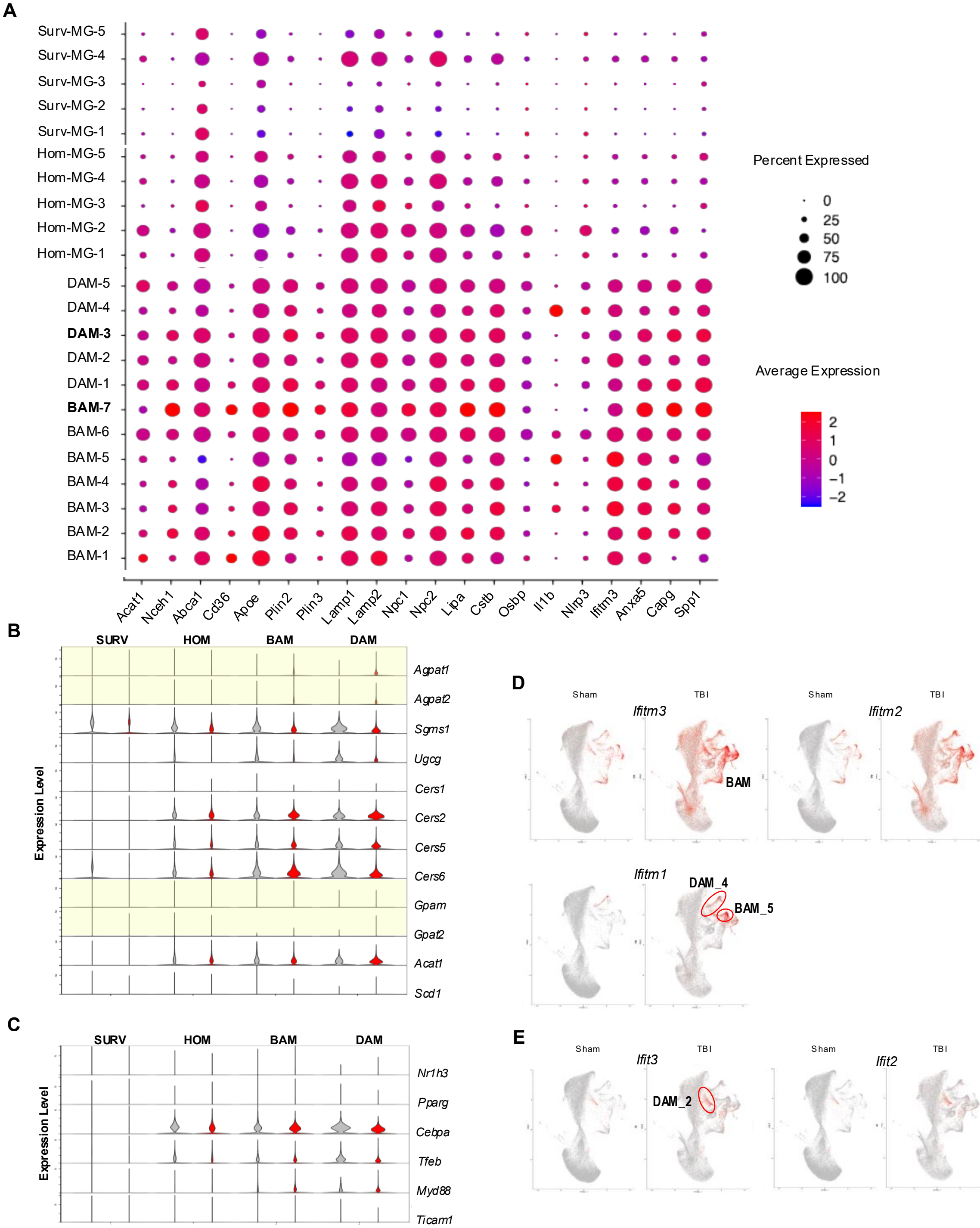
A
Surv-MG-5
Surv-MG-4
Surv-MG-3
Surv-MG-2
Surv-MG-1
Hom-MG-5
Hom-MG-4
Hom-MG-3
Hom-MG-2
Hom-MG-1
DAM-5
DAM-4
DAM-3
DAM-2
DAM-1
BAM-7
BAM-6
BAM-5
BAM-4
BAM-3
BAM-2
BAM-1
Acat1
Nceh1
Abca1
Cd36
Apoe
Plin2
Plin3
Lamp1
Lamp2
Npc1
Npc2
Lipa
Cstb
Osbp
Il1b
Nlrp3
Ifitm3
Anxa5
Capg
Spp1
Percent Expressed
0
25
50
75
100
Average Expression
2
1
0
−1
−2
B
SURV
HOM
BAM
DAM
Expression Level
Agpat1
Agpat2
Sgms1
Ugcg
Cers1
Cers2
Cers5
Cers6
Gpam
Gpat2
Acat1
Scd1
C
SURV
HOM
BAM
DAM
Expression Level
Nr1h3
Pparg
Cebpa
Tfeb
Myd88
Ticam1
D
Sham
TBI
Ifitm3
BAM
Sham
TBI
Ifitm2
Ifitm1
DAM_4
BAM_5
E
Sham
TBI
Ifit3
DAM_2
Sham
TBI
Ifit2

Figure S3

Figure S3. Lipid reprogramming is more pronounced in lipid-accumulating microglia and macrophages. Related to Figures 2 and 3.
(A) Dot plot showing expression of selected lipid metabolism, lysosomal function and phagocytosis marker genes across major microglial and macrophage subtypes. Each dot represents the expression of a given gene (x-axis) in each cluster (y-axis) of all four main categories**:** homeostatic microglia (HOM), surveillance microglia (SURV), disease-associated microglia (DAM), and border-associated macrophages (BAM). Dot size indicates the percentage of cells in each cluster expressing specified gene; color intensity reflects the average scaled expression level (z-score) among expressing cells. (B) Violin plots (generated by ggplot() in Seurat) showing expression levels of lipid synthesis genes involved in the synthesis of triglycerides (highlighted in yellow: *Agpat1, Agpat2, Gpam*, and *Gpat*), sphingomyelin (*Sgms1*), ceramides (*Cers1*, 2, 5, and 6) and cholesterol (*Acat1* and *Scd1*). (C) Violin plots (generated by ggplot() in Seurat) showing expression levels of select transcription factors involved in lipid metabolism and TLR signaling. (D-E) Feature plot visualization of interferon-stimulated gene expression across all microglial and monocyte populations emphasizing the heterogeneous and cluster-specific activation of interferon-responsive genes following TBI. (D) *Ifitm3* and *Ifitm2* show elevated expression in both BAMs and DAMs, with a stronger signal in BAM subclusters. *Ifitm1* is primarily expressed in *DAM4* and *BAM5*, highlighting a subpopulation-specific interferon response. (E) *Ifit3* and *Ifit2* exhibit highly selective expression in *DAM2*.

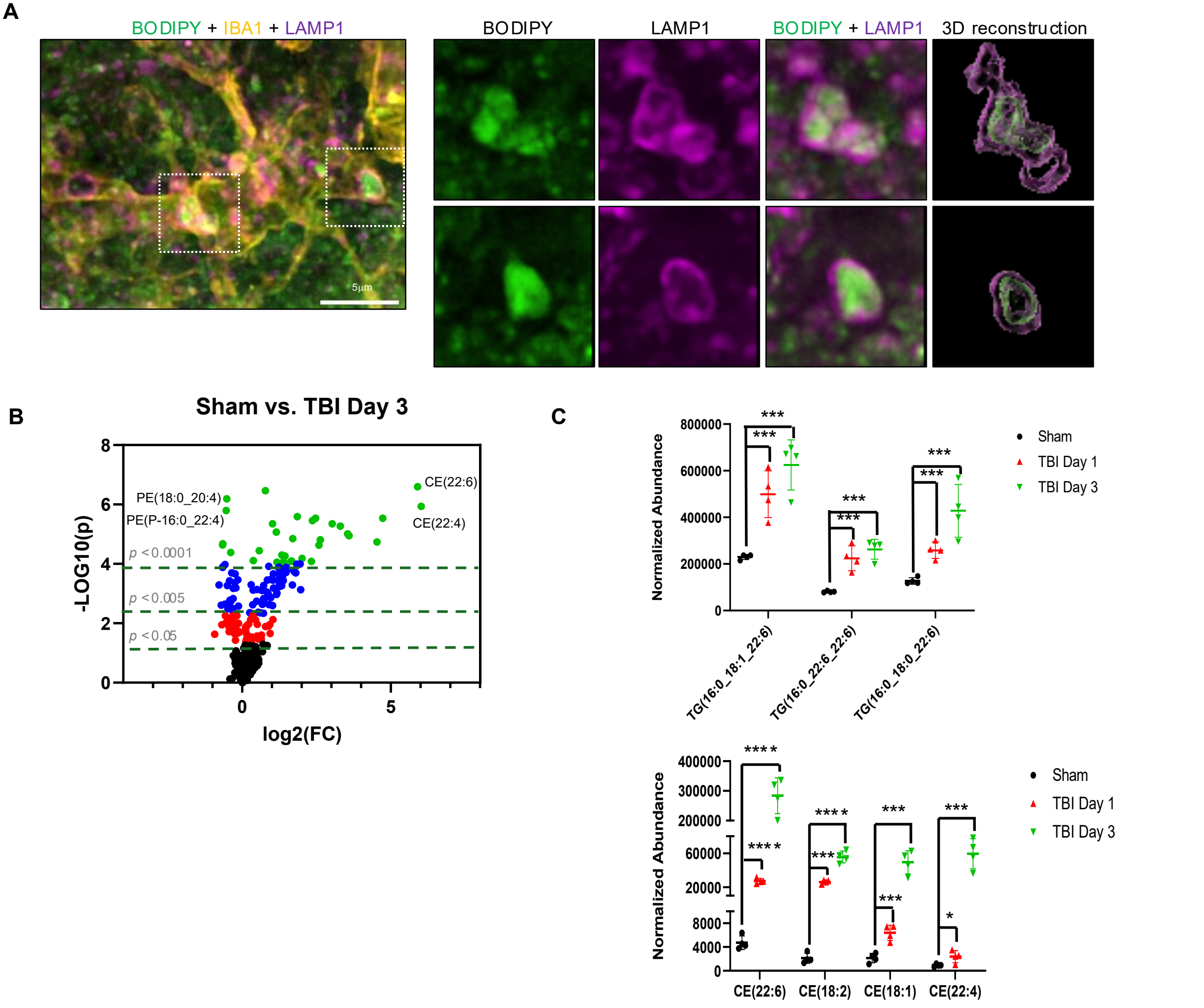

Figure S4. Lipid accumulates in the lysosomes of microglia and macrophages after TBI. Related to Figure 4. (A) Close up confocal image of active microglia after TBI and insets of single channels used for 3D reconstruction. (B) MetaboAnalyst generated volcano plot highlighting lipids with P < 0.05 (red), P < 0.005 (blue), and P < 0.0001 (green, based on t-test) in TBI (day 3) vs sham. Identity of selected lipid species is indicated. n = 4 mice/group. (C) Normalized abundance of selected differentially abundant triglyceride (TG, top) and cholesteryl ester (CE, bottom) species across experimental groups. Each point represents an individual sample; bars indicate mean ± standard deviation. Lipid species shown on the x-axis are among those with the highest fold-change and statistical significance in TBI vs Sham groups. * p <0.05, ** p < 0.01, *** p < 0.005, **** p < 0.0001 (one-way ANOVA with FDR correction).

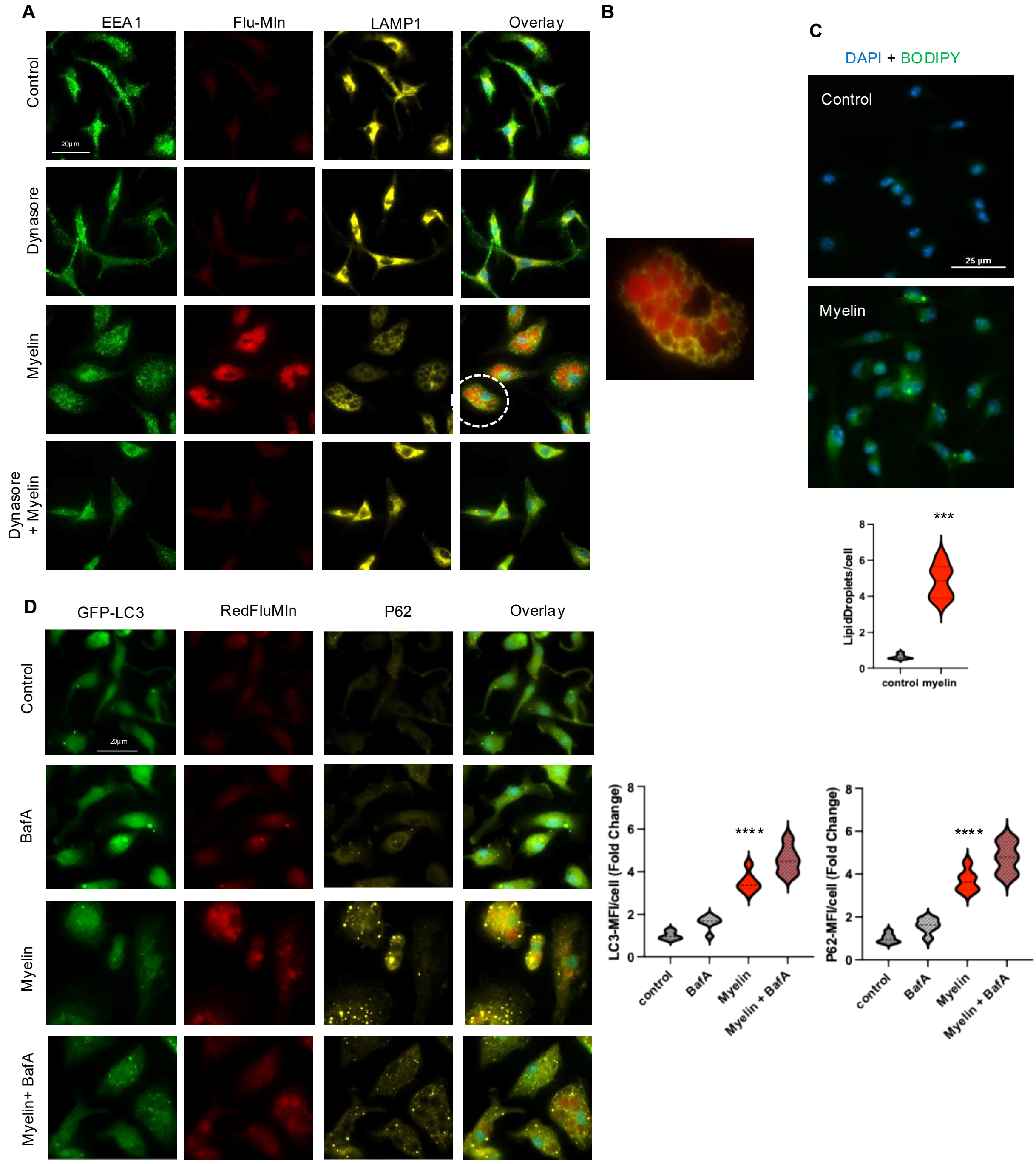
A
EEA1
Flu-MIn
LAMP1
Overlay
Control
Dynasore
Myelin
Dynasore + Myelin
20μm
B
C
DAPI + BODIPY
Control
25 μm
Myelin
***
LipidDroplets/cell
control
myelin
D
GFP-LC3
RedFluMIn
P62
Overlay
Control
BafA
Myelin
Myelin+ BafA
20μm
LC3-MFI/cell (Fold Change)
P62-MFI/cell (Fold Change)
****
control
BafA
Myelin
Myelin + BafA

Figure S5

Figure S5. Myelin phagocytosis inhibits lysosomal function and autophagy after TBI. Related to Figure 5. (A) Immunofluorescence images demonstrating phagocytic myelin uptake and its subcellular localization to lysosomes in wild-type bone marrow–derived macrophages (BMDMs). BMDM were treated with vehicle (control), purified mouse myelin (100 µg/mL, 4 hr), Dynasore (10 µM, 4.5 hr), or Dynasore pre-treatment (30 min) followed by myelin (4 hr). Cells were stained for early endosomes (EEA1, green), phagocytosed myelin (RedFluoroMyelin, red), and lysosomes (LAMP1, yellow). BMDMs exhibited robust myelin uptake, which was prevented by Dynasore, confirming that myelin internalization is dynamin-dependent. (B) Inset from indicated are in panel A, demonstrating accumulation of myelin (RedFluoroMyelin) within LAMP1 lysosomes. (C) Images and quantification demonstrating that myelin treatment induced neutral lipid accumulation in wild-type BMDMs. BMDM were treated with vehicle (control) or myelin (100 µg/mL, 24 hr). $P < 0.05$ (unpaired *t*-test). (D) Immunofluorescence images and quantification demonstrating inhibition of autophagy flux in GFP-LC3–expressing BMDMs treated with myelin. GFP-LC3 BMDM were treated with vehicle or myelin (100 µg/mL, 24 hr), ± Bafilomycin A1 (50 nM for the final 4 hr). ****$P < 0.001$ vs control; values are mean ± SEM, n = 6 (3 replicates of 2 independent experiments); **** $P < 0.0001$ (two-way ANOVA with Tukey's).

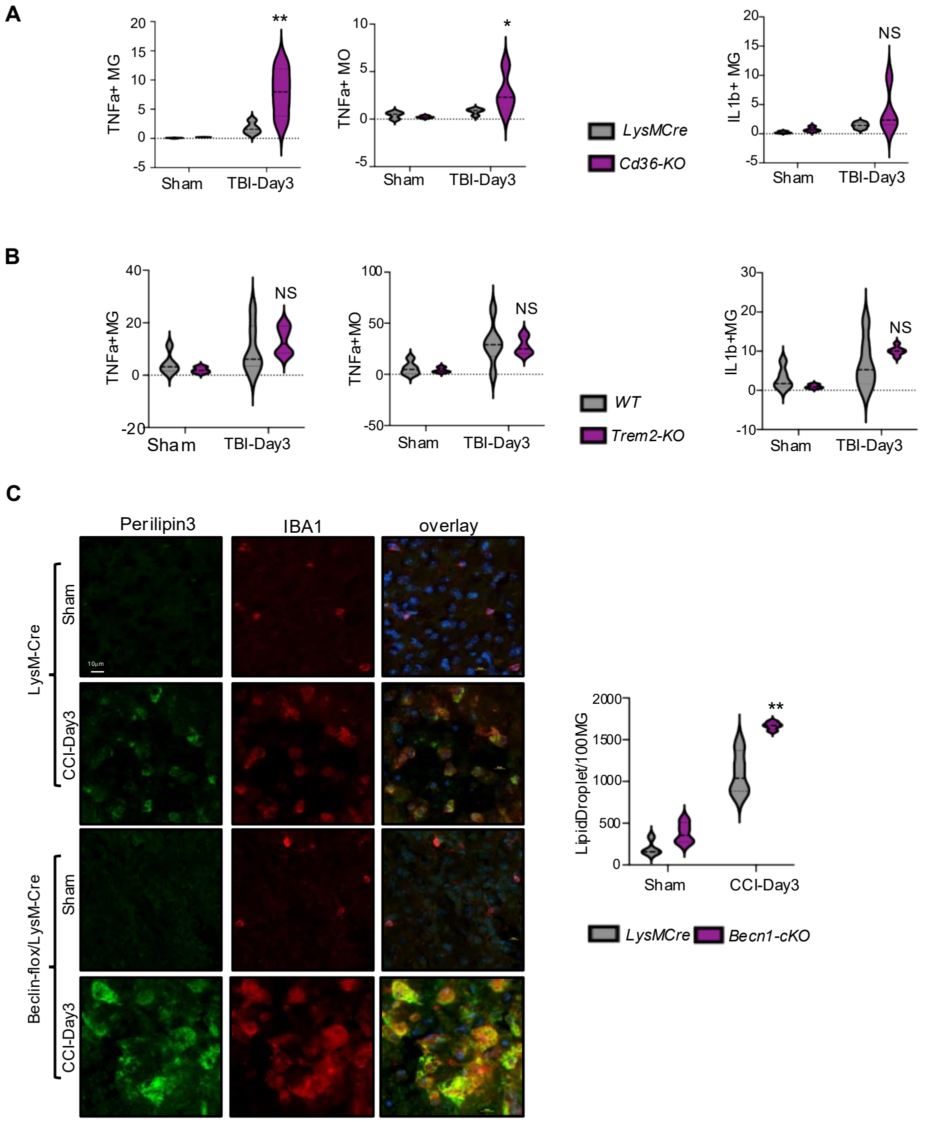

Figure S6. Autophagy deficiency exacerbates lipid accumulation and lipid metabolism reprograming. Related to Figures 5 and 6. (A-B) Flow cytometry quantification of inflammatory cytokine TNFa (left) and IL1b (right) expression in microglia and monocytes from *Cd36*- (A) and *Trem2*- (B) deficient as compared to corresponding WT control mice following TBI. n = 6 mice/group; *P < 0.05, **P < 0.01 vs corresponding WT (two-way ANOVA with Tukey's). (C) Immunofluorescence images and quantification of demonstrating increased lipid droplet (Perilipin 3) formation in microglia/monocytes (IBA1) in *Beclin1-cKO* vs *LysMCre (WT)* mouse brains at day 3 post-TBI. n = 4 mice/group; **P < 0.01 vs *LysMCre* (WT) (two-way ANOVA with Tukey's).
